# Supplementary figures and images for: Evolutionary Analysis of Transcriptional Regulation Mediated by Cdx2 in Rodents
Source: Cell Prolif. 2025 Jul 29;59(3):e70103. doi: 10.1111/cpr.70103 (PMC12961547; doi:10.1111/cpr.70103)

Fig S1

A

DNA Binding Domain

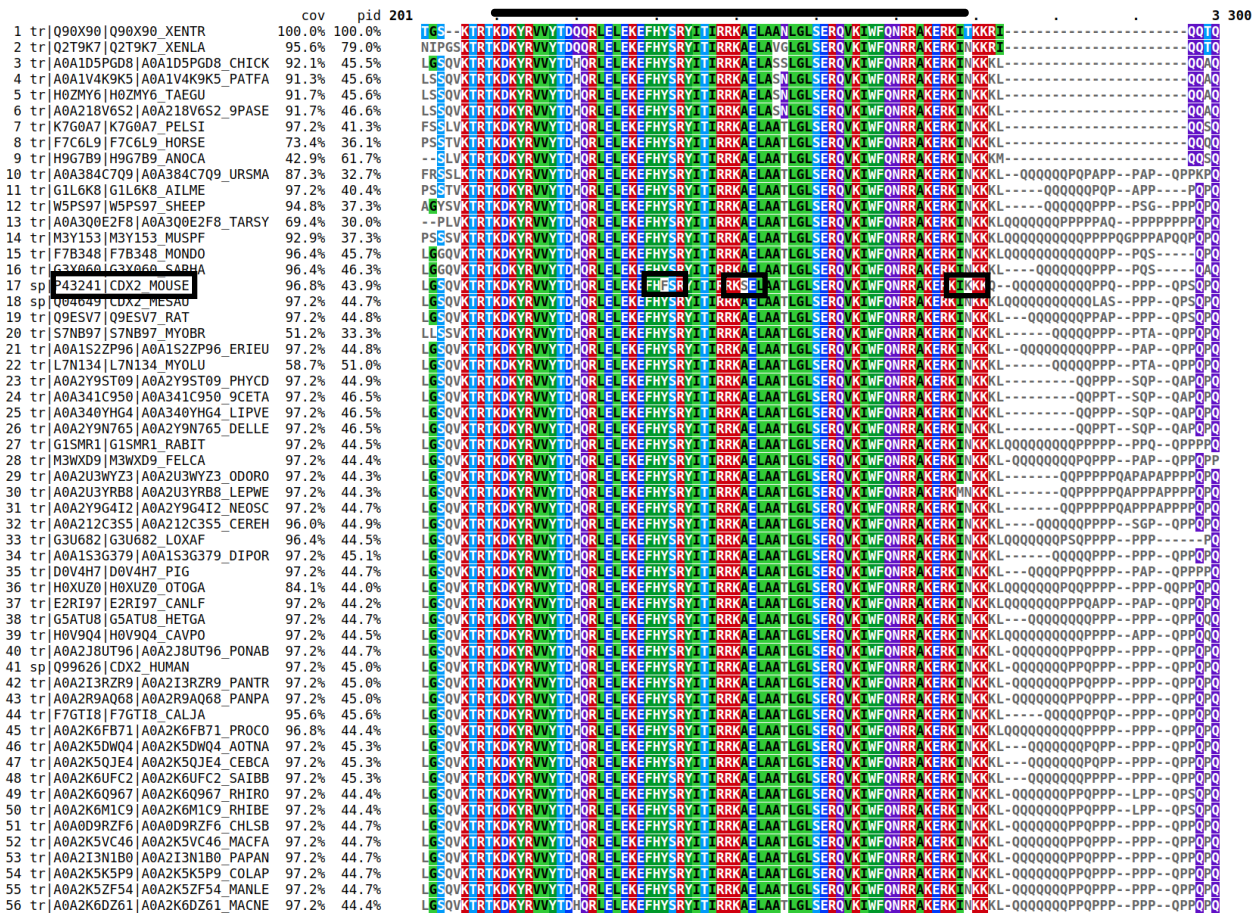

B

DNA Binding Domain

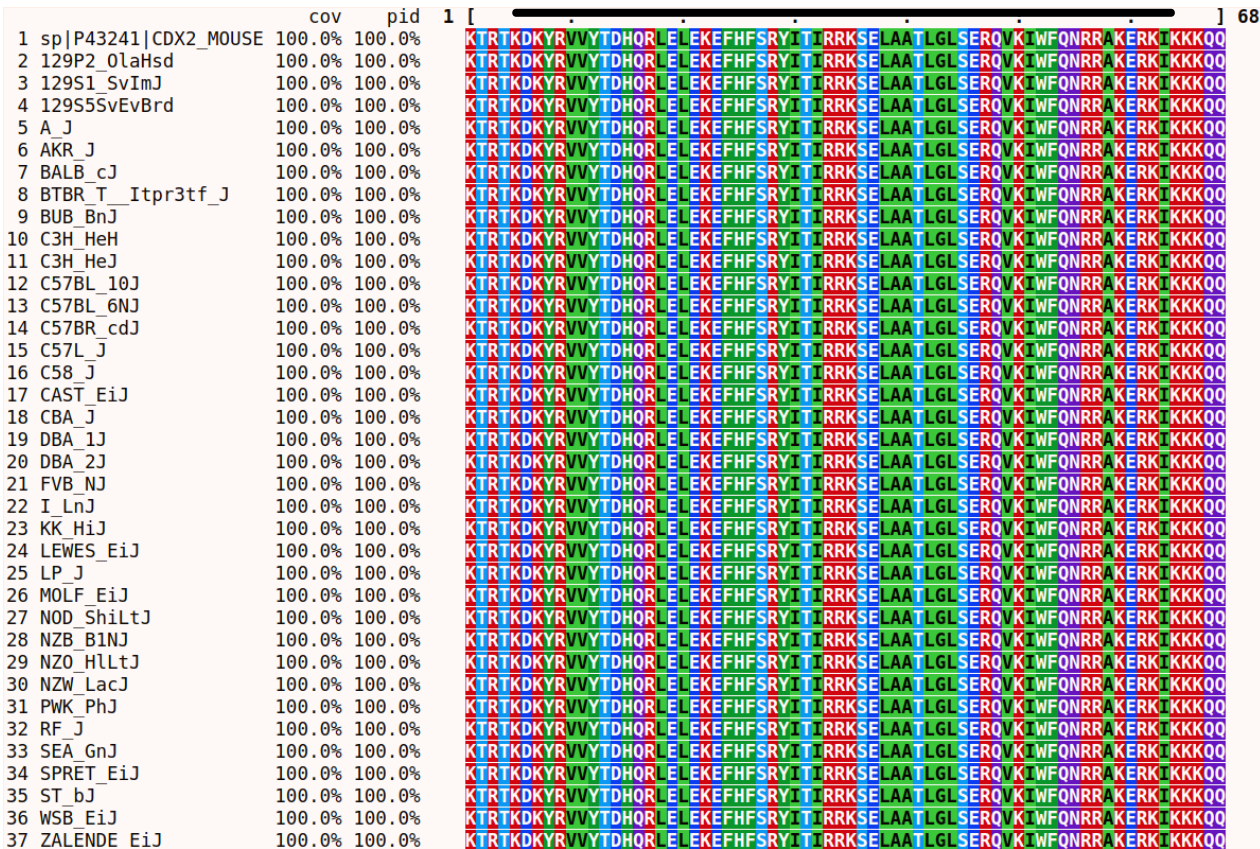

Fig S2

A

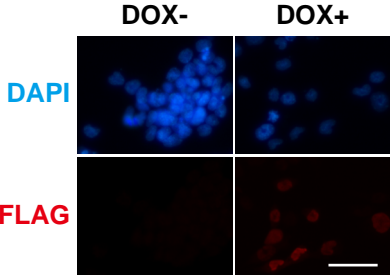

B

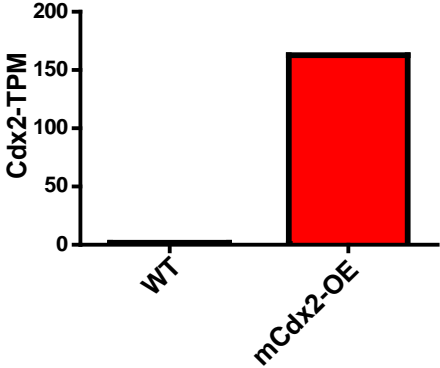

C

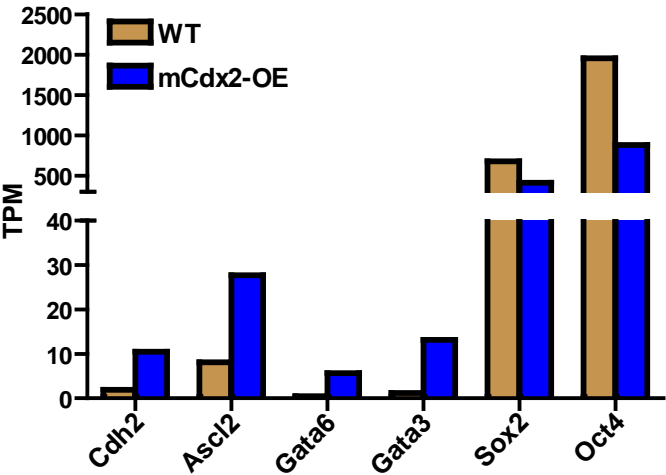

D

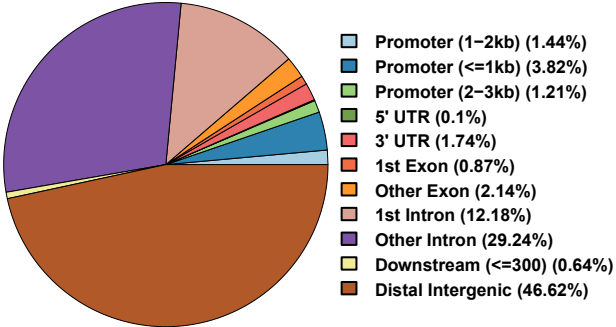

**Fig S3**

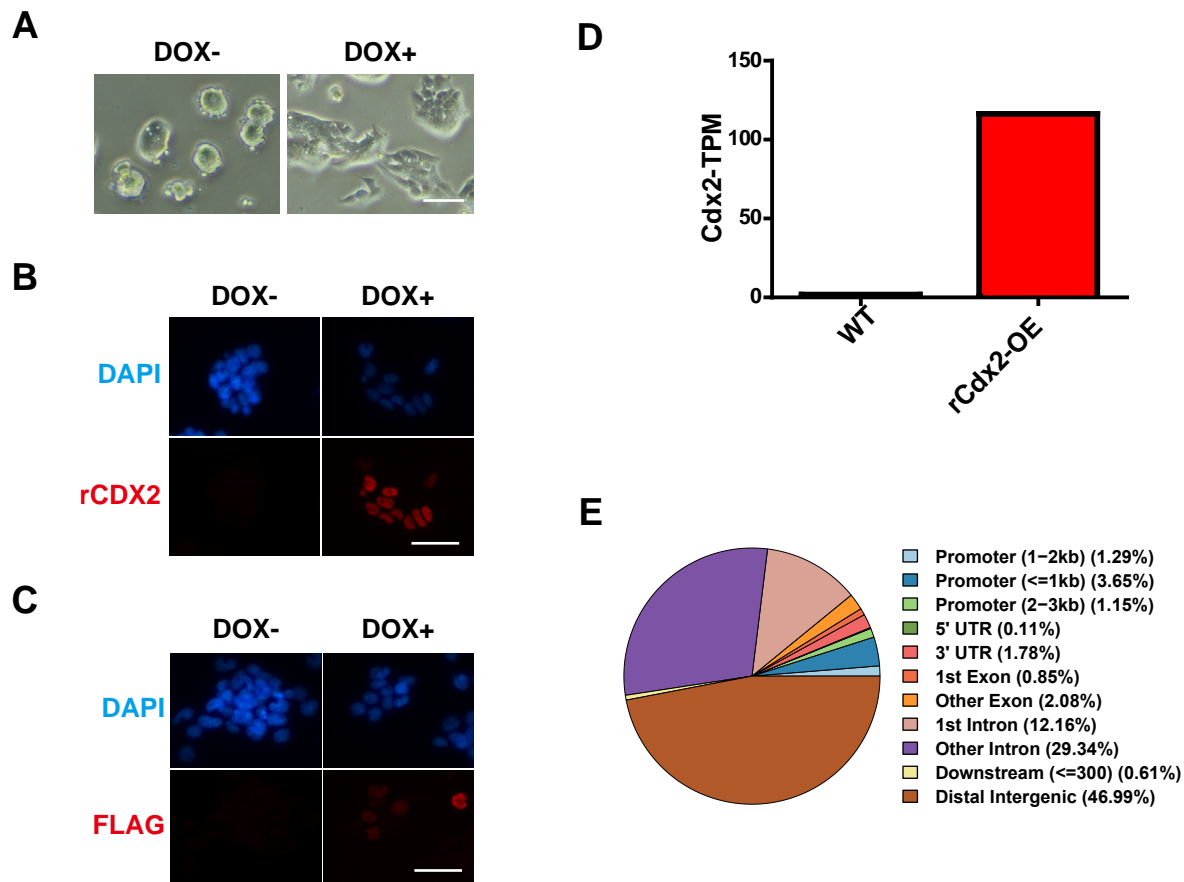

**Fig S4**

**A**

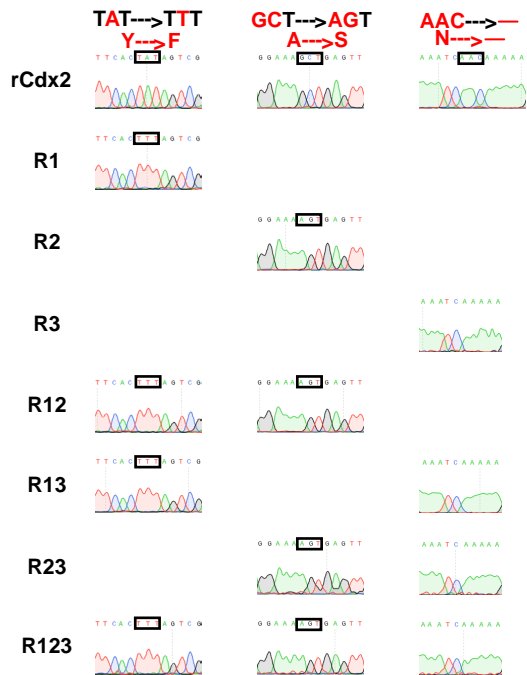

**B**

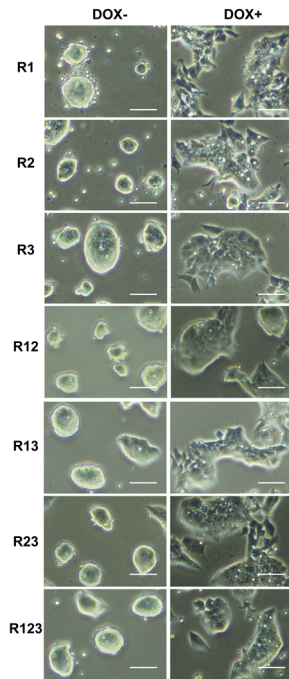

**C**

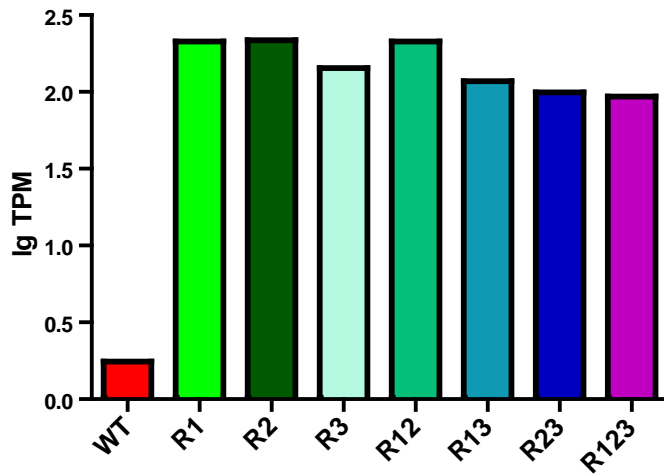

**Fig S5**

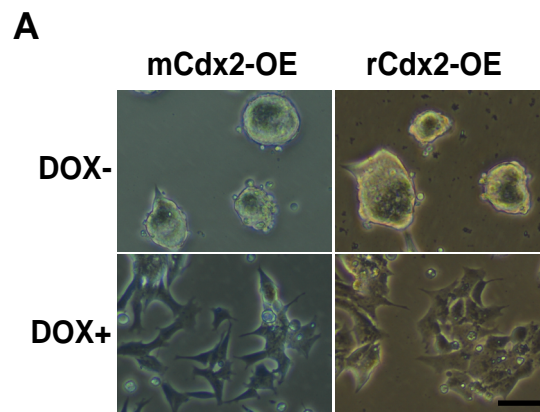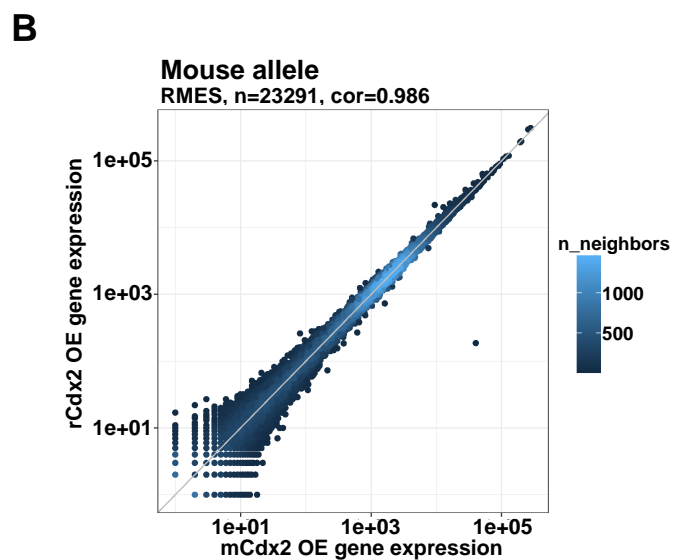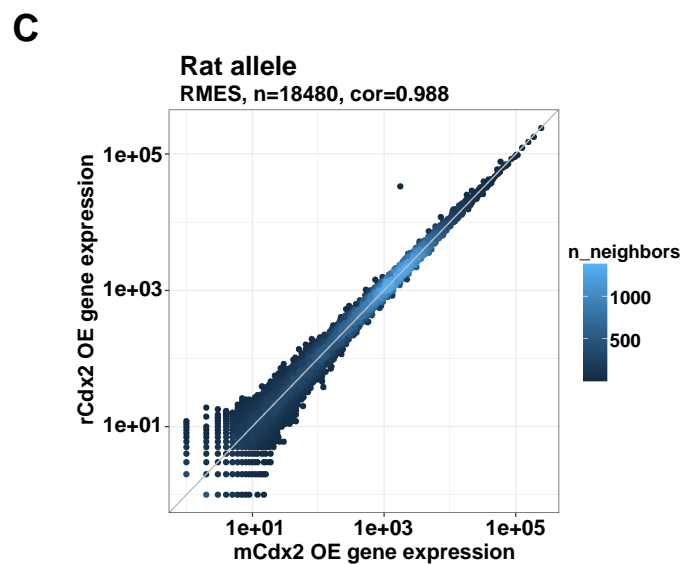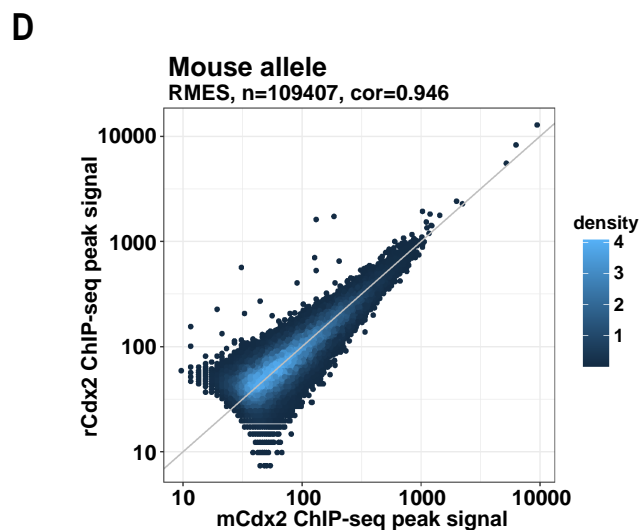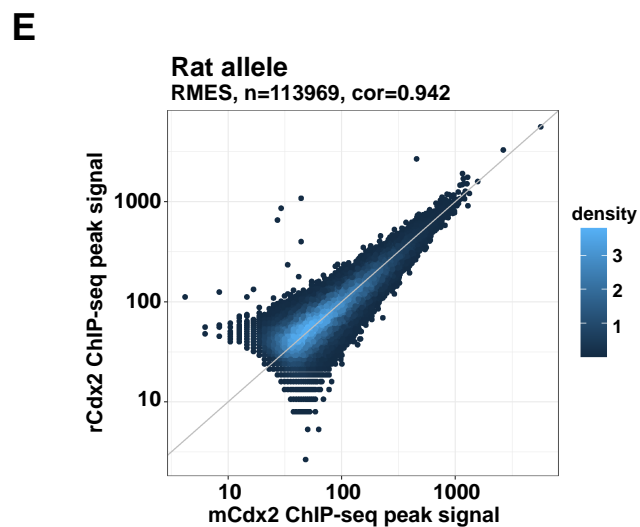

Fig S6

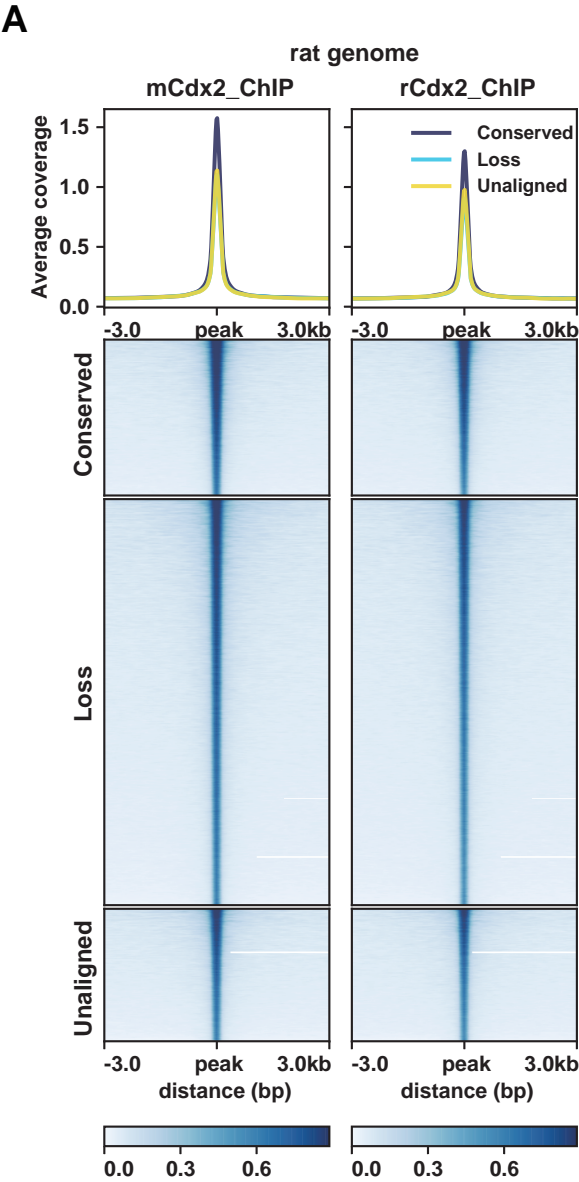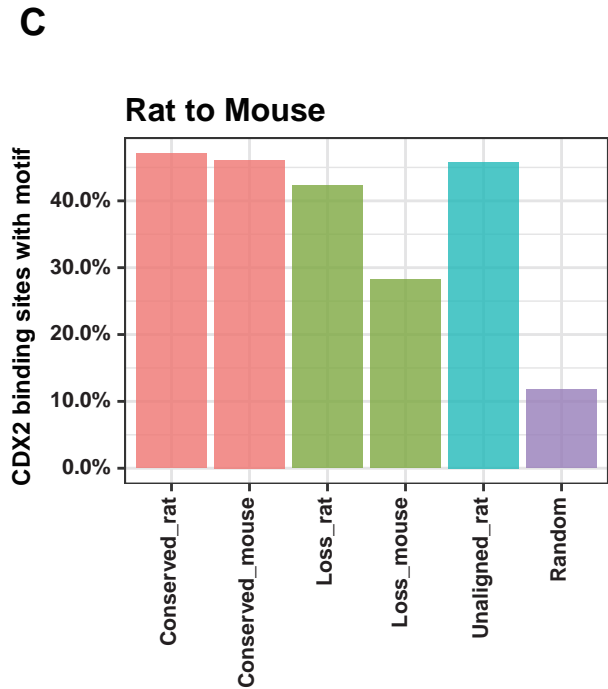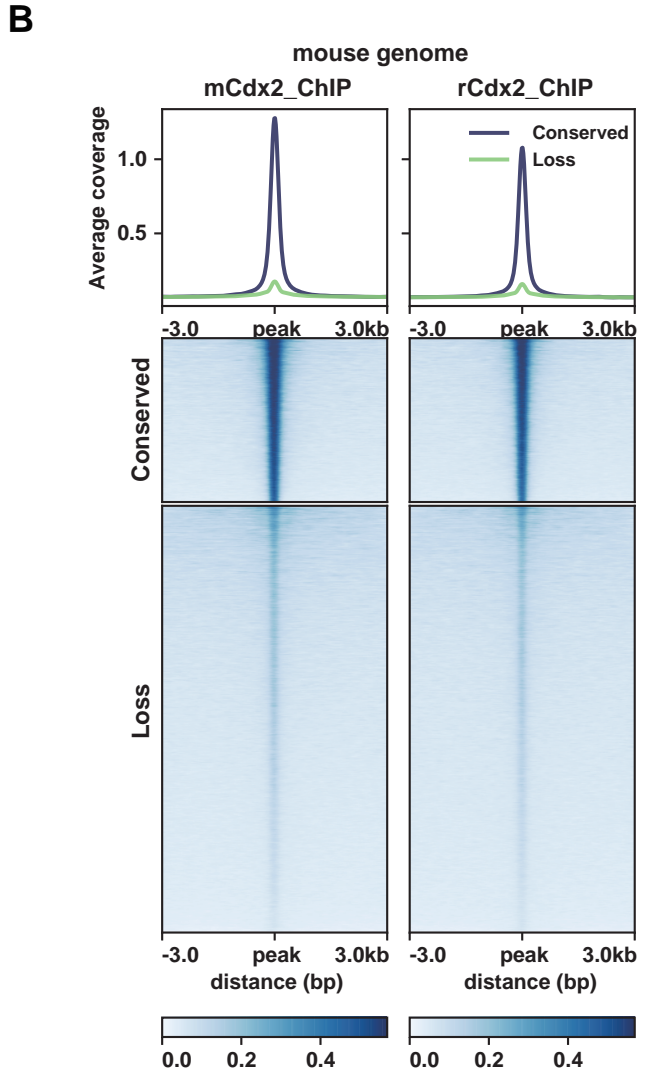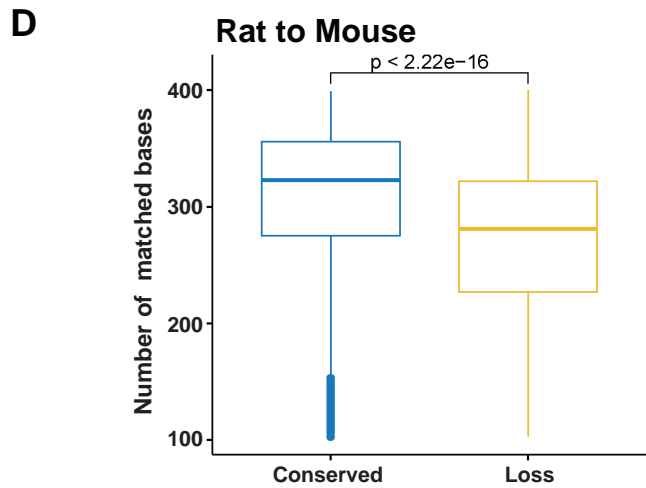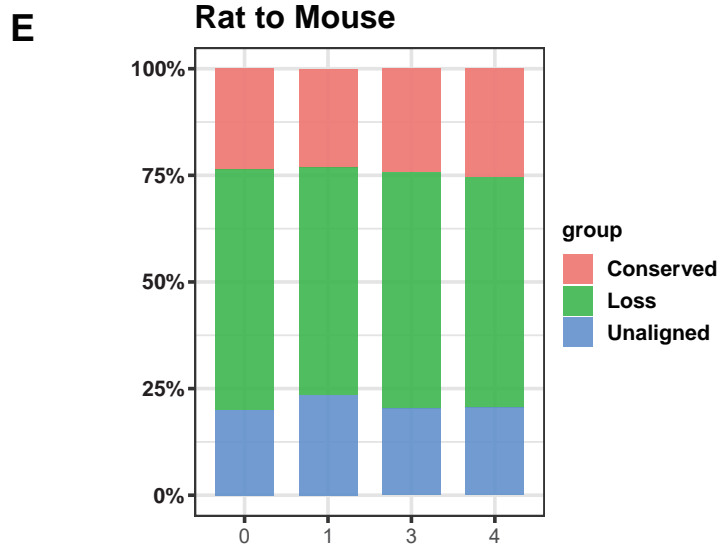

Figure S7

A

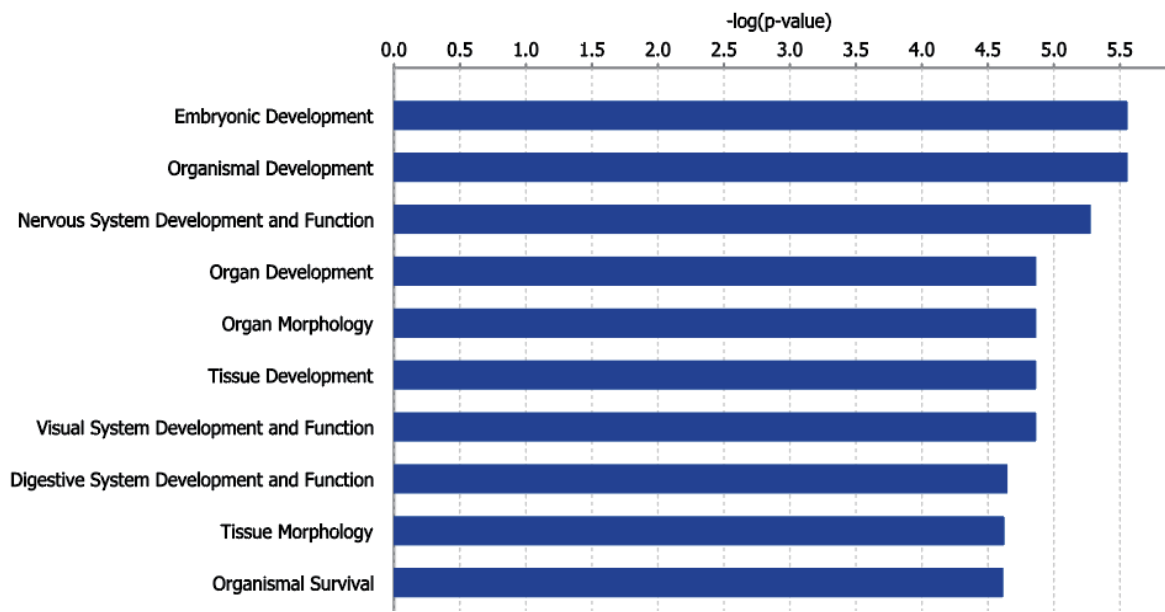

B

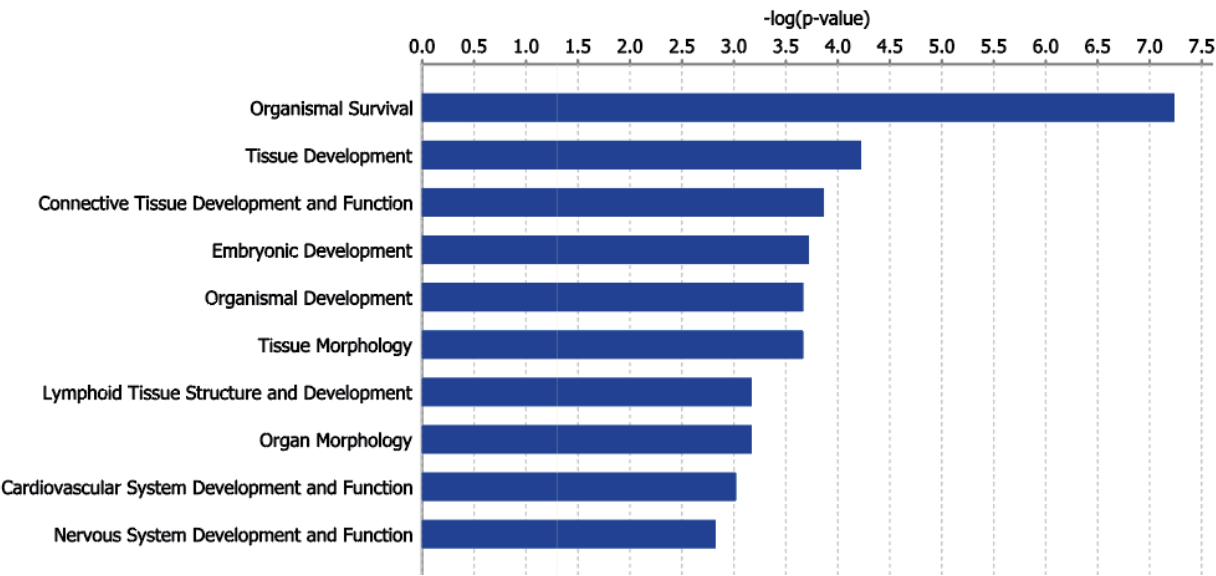

Supplement: Supplementary file 1 — Figure S1. mCdx2 has three specific amino acid changes in the DNA Binding Domain. (A) Sequence alignment of more species showed the three specific amino acid changes in the DBD were exclusively found in mouse. (B) Sequence alignment of all mouse strains with available genome further indicated the three amino acid changes were mouse specific. Figure S2. mCdx2 is an important regulator of ES cell differentiation. (A) Immunofluorescence (IF) experiments confirmed the successful expression of mCdx2 using the antibody against FLAG. (B) RNA‐seq results indicated the successful overexpression of mCdx2. (C) Gene expression data showed upregulation of TSC‐related genes and downregulation of pluripotency‐related genes. (D) mCdx2‐ChIP peak distribution on the genome. Figure S3. Establishment of rCdx2 stably expressed ESC lines. (A) The cell morphology changes after DOX induction. (B) Immunofluorescence (IF) experiments confirmed the successful expression of rCdx2 using the antibody against CDX2. (C) Immunofluorescence (IF) experiments confirmed the successful expression of rCdx2 using the antibody against FLAG. (D) The RNA‐seq results indicated the successful overexpression of rCdx2. (E) rCdx2‐ChIP peak distribution on the genome. Figure S4. Establishment of Cdx2 mutants stably expressed ESC lines. (A) Verification of Cdx2 mutants by Sanger sequencing. (B) The morphology changes after DOX treatment transfected with Cdx2 mutants. (C) The gene expression results confirmed the successful overexpression of Cdx2 mutants. Figure S5. mCdx2 and rCdx2 are conserved at the molecular level. (A) The morphology of RMES cells changed after mCdx2 and rCdx2 induction. (B) Comparing gene expression change of mouse allele between mCdx2‐OE RMES cells and rCdx2‐OE RMES cells. (C) Comparing gene expression change of rat allele between mCdx2‐OE RMES cells and rCdx2‐OE RMES cells. (D) Comparing ChIP‐peak signal on mouse allele between mCdx2‐OE RMES cells and rCdx2‐OE RMES cells. (E) Comparing ChIP‐p [file CPR-59-e70103-s002.pdf]
