# Supplementary material for: Evolutionary Analysis of Transcriptional Regulation Mediated by Cdx2 in Rodents
Source: Cell Prolif. 2025 Jul 29;59(3):e70103. doi: 10.1111/cpr.70103 (PMC12961547; doi:10.1111/cpr.70103)
Supplement: Supplementary file 2 — Table S1. The number of three types of peaks located at proximal and distal regions from mouse to rat direction. Table S2. The number of three types of peaks located at proximal and distal regions from rat to mouse direction. Table S3. Sample sequencing details. [file CPR-59-e70103-s001.docx]

**Evolutionary Analysis of Transcriptional Regulation Mediated by Cdx2 in Rodents**

Weizheng Liang^1,2,3,10^, Guipeng Li^2,3,4,10^, Yukai Wang^5,6,7,8,10^, Wencheng Wei^2,3^, Rui Chen^2,3^, Siyue Sun^2,3^, Diwen Gan^2,3^, Hongyang Yi^2,3^, Bernhard Schaefke^2,3,4^, Yuhui Hu^2,3,4^, Qi Zhou^5,6,7,8,9^, Wei Li^5,6,7,9^, Huanhuan Cui^2,3,4*^, Wei Chen^2,3,4*^

1. Central Laboratory, The First Affiliated Hospital of Hebei North University, Zhangjiakou 075000, Hebei, China
2. Shenzhen Key Laboratory of Gene Regulation and Systems Biology, School of Life Sciences, Southern University of Science and Technology, Shenzhen 518055, China
3. Department of Systems Biology, School of Life Sciences, Southern University of Science and Technology, Shenzhen 518055, China.
4. Academy for Advanced Interdisciplinary Studies, Southern University of Science and Technology, Shenzhen 518055, China
5. State Key Laboratory of Stem Cell and Reproductive Biology, Institute of Zoology, Chinese Academy of Sciences, Beijing, China
6. Institute for Stem Cell and Regeneration, Chinese Academy of Sciences, Beijing, China
7. Department of National Stem Cell Resource Center, Institute of Zoology, Chinese Academy of Sciences, Beijing, China
8. Beijing Institute for Stem Cell and Regenerative Medicine, Beijing, China
9. University of Chinese Academy of Sciences, Beijing, China
10. These authors contributed equally to this work.

* Correspondence: chenw@sustech.edu.cn (WC), cuihh@sustech.edu.cn (HHC)

**Figure S1. mCdx2 has three specific amino acid changes in the DNA Binding Domain**

(A) Sequence alignment of more species showed the three specific amino acid changes in the DBD were exclusively found in mouse. (B) Sequence alignment of all mouse strains with available genome further indicated the three amino acid changes were mouse specific.

**Figure S2. mCdx2 is an important regulator of ES cell differentiation**

(A) Immunofluorescence (IF) experiments confirmed the successful expression of mCdx2 using the antibody against FLAG. (B) RNA-seq results indicated the successful overexpression of mCdx2. (C) Gene expression data showed upregulation of TSC-related genes and downregulation of pluripotency-related genes. (D) mCdx2-ChIP peak distribution on the genome.

**Figure S3. Establishment of rCdx2 stably expressed ESC lines**

(A) The cell morphology changes after DOX induction. (B) Immunofluorescence (IF) experiments confirmed the successful expression of rCdx2 using the antibody against CDX2. (C) Immunofluorescence (IF) experiments confirmed the successful expression of rCdx2 using the antibody against FLAG. (D) The RNA-seq results indicated the successful overexpression of rCdx2. (E) rCdx2-ChIP peak distribution on the genome.

**Figure S4. Establishment of Cdx2 mutants stably expressed ESC lines**

(A) Verification of Cdx2 mutants by Sanger sequencing. (B) The morphology changes after DOX treatment transfected with Cdx2 mutants. (C) The gene expression results confirmed the successful overexpression of Cdx2 mutants.

**Figure S5. mCdx2 and rCdx2 are conserved at the molecular level**

(A) The morphology of RMES cells changed after mCdx2 and rCdx2 induction. (B) Comparing gene expression change of mouse allele between mCdx2-OE RMES cells and rCdx2-OE RMES cells. (C) Comparing gene expression change of rat allele between mCdx2-OE RMES cells and rCdx2-OE RMES cells. (D) Comparing ChIP-peak signal on mouse allele between mCdx2-OE RMES cells and rCdx2-OE RMES cells. (E) Comparing ChIP-peak signal on rat allele between mCdx2-OE RMES cells and rCdx2-OE RMES cells.

**Figure S6. Species-specific binding of Cdx2 and its effects on gene expression**

(A) The heatmap of signal intensity of the three peak types at rat genome and (B) after being aligned to the mouse genome. (C) The percentage of Cdx2 binding sites with motif in three peak types from rat to mouse direction (D) The distribution of mapped bases in the conserved and loss peaks from rat to mouse direction. (E) The ratio of different types of peaks in the four types of genes with different regulatory pattern from rat to mouse direction.

**Figure S7.** Gene ontology (GO) enrichment analysis using Ingenuity Pathway Analysis (IPA) software (QIAGEN) for type 1 gene list (A) and type 3 gene list (B)

**Table S1. The number of three types of peaks located at proximal and distal regions from mouse to rat direction**

| m2r | total | proximal | distal |
| --- | --- | --- | --- |
| unaligned | 13580 | 512 | 13068 |
| conserved | 17321 | 726 | 16595 |
| loss | 41430 | 1915 | 39515 |

**Table S2. The number of three types of peaks located at proximal and distal regions from rat to mouse direction**

| r2m | total | proximal | distal |
| --- | --- | --- | --- |
| unaligned | 14715 | 368 | 14347 |
| conserved | 17317 | 461 | 16856 |
| loss | 45105 | 1382 | 43723 |

**Table S3: Sample sequencing details**

| Sample name | method | source name | Replicate |
| --- | --- | --- | --- |
| WT mESC | **RNA-seq** | **mESC** | **2** |
| mCDX2 OE in mESC | **RNA-seq** | **mESC** | **2** |
| rCDX2 OE in mESC | **RNA-seq** | **mESC** | **2** |
| rCDX2_R1 OE in mESC | **RNA-seq** | **mESC** | **2** |
| rCDX2_R2 OE in mESC | **RNA-seq** | **mESC** | **2** |
| rCDX2_R3 OE in mESC | **RNA-seq** | **mESC** | **2** |
| rCDX2_R12 OE in mESC | **RNA-seq** | **mESC** | **2** |
| rCDX2_R13 OE in mESC | **RNA-seq** | **mESC** | **2** |
| rCDX2_R23 OE in mESC | **RNA-seq** | **mESC** | **2** |
| rCDX2_R123 OE in mESC | **RNA-seq** | **mESC** | **2** |
| WT RMES | **RNA-seq** | **RMES** | **2** |
| mCDX2 OE in RMES | **RNA-seq** | **RMES** | **1** |
| rCDX2 OE in RMES | **RNA-seq** | **RMES** | **1** |
| WT mESC | **ATAC-seq** | **mESC** | **1** |
| mCDX2 OE in mESC | **ATAC-seq** | **mESC** | **1** |
| mCDX2 OE in mESC | **ChIP-seq** | **mESC** | **1** |
| rCDX2 OE in mESC | **ChIP-seq** | **mESC** | **1** |
| mCDX2 OE in RMES | **ChIP-seq** | **RMES** | **1** |
| rCDX2 OE in RMES | **ChIP-seq** | **RMES** | **1** |
